# Efficient and promising oxidative desulfurization of fuel using Fenton like deep eutectic solvent

**DOI:** 10.1038/s41598-024-62781-x

**Published:** 2024-06-01

**Authors:** Fatemeh Armandsefat, Sholeh Hamzehzadeh, Najmedin Azizi

**Affiliations:** Chemistry and Chemical Engineering Research Center of Iran, P.O. Box 14335-186, Tehran, Iran

**Keywords:** Deep oxidative desulfurization, Deep eutectic solvent, Fenton-like, Dibenzothiophene, Environmental sciences, Natural hazards, Chemistry

## Abstract

Oxidative desulfurization (ODS) has emerged as a prominent technique for the removal of sulfur compounds from fuels, aiming to comply with stringent environmental regulations and minimize sulfur dioxide emissions. Herein, Fenton-like deep eutectic solvents (DESs) were synthesized as a catalyst and reaction medium and their application for the ODS process was investigated. The study encompassed the optimization of DES composition, reaction conditions, and the influence of different parameters on the desulfurization efficiency. The experimental findings demonstrated that the Fenton-like DES exhibited outstanding catalytic activity in the oxidative desulfurization of fuel. The optimized conditions involved conducting the reaction at room temperature for 2.5 h, using 200 mg of the prepared DES (HNFM-FeCl_4_) as both the extraction solvent and catalyst. An oxidant-to-sulfur (O/S) ratio of approximately 3:1 was maintained, with a 30 wt% H_2_O_2_ solution utilized as the oxidant. The analysis of the reaction products using GC–MS revealed a remarkable yield of 98% for dibenzothiophene sulfone. The DES provided a suitable medium for the reaction, enhancing the solubility and availability of sulfur compounds. The iron catalyst, in the presence of hydrogen peroxide, facilitated the oxidation of sulfur-containing compounds to their corresponding sulfones, which can be easily separated from the fuel phase. The DES catalysts exhibited stability and recyclability, making them suitable for practical applications in fuel desulfurization processes.

## Introduction

**Figure 1:**
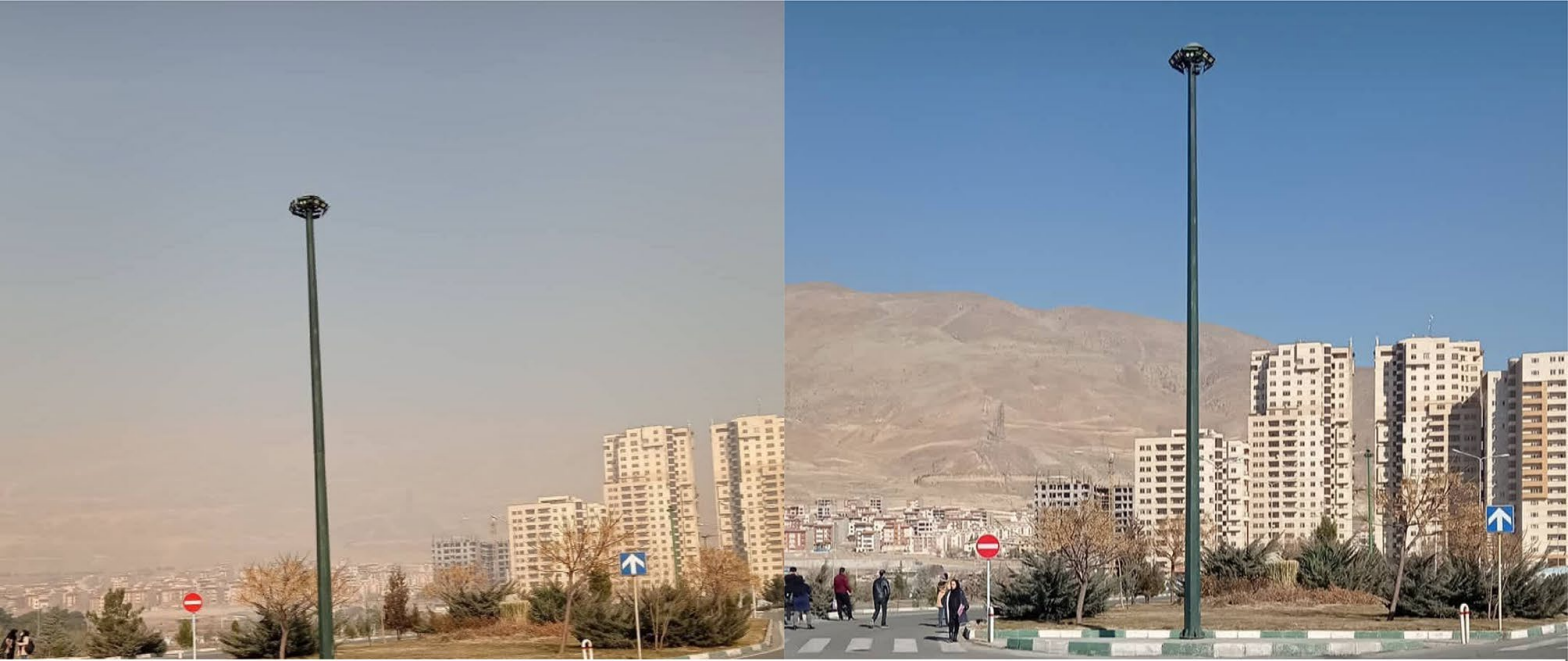
Left: a polluted day in west of Tehran on January 12th 2021, right: a clear day in west of Tehran on January 16th 2021.

Over the past two decades, the primary challenges faced by society have been global warming and air pollution^1^. In response, politicians have implemented stringent laws, and researchers have made advancements in materials and techniques to reduce emissions of greenhouse gases, NO_x_, and SO_x_. It has been determined that the combustion of diesel fuel is a major contributor to SO_x_ gas emissions. As a first step, many countries have successfully reduced the sulfur content in diesel fuel to ten parts per million (ppm) by 2009^2,3^, and now they strive to achieve the use of no-sulfur fuels. The emission of SOx gases has been associated with various harmful effects, including acid rain^4^, pollution^5^, and the formation of photochemical smog^6^ in densely populated cities such as Tehran (as illustrated in Fig. 1), Mumbai, and others.

Hydrodesulfurization (HDS) is a traditional hydrotreating method widely employed in desulfurization units to eliminate mercaptans, disulfides, sulfides, and light thiophenic sulfur from oils and petroleum fuels^7^. HDS necessitates specific catalysts, such as Ni^8^, Cu^8^, Mo^9^, and Co^10^, supported on Al_2_O_3_. However, HDS has certain drawbacks, including the need for harsh reaction conditions like high temperatures (563–728 K) and high hydrogen pressures (10.2–204.1 atm)^11^. Moreover, the steric hindrance of polycyclic organic sulfides hinders their desulfurization using HDS^12^. Researchers have explored alternative methods to address these challenges, such as oxidative desulfurization (ODS)^13,14^. ODS is an alternative method to HDS that has gained attention in recent decades^15,16^. ODS offers a solution to overcome these challenges. In ODS, the sulfur compounds are selectively oxidized to sulfoxides or sulfones, which can be easily separated from the fuel phase^17,18^. One of the drawbacks of traditional ODS methods is using organic solvents as the extracting phase^19^. These solvents often have toxicity, volatility, flammability, and non-eco-friendly limitations^20^. Researchers have turned to green solvents such as ionic liquids (ILs) and DESs as promising alternative to address these concerns.

DESs are a type of ionic liquid-like solvent that exhibit unique properties and have gained attention in various fields of research and industrial applications. DES are formed by the combination of a hydrogen bond donor (HBD), such as a quaternary ammonium salt or a carboxylic acid, and a hydrogen bond acceptor (HBA), such as a metal salt or a Lewis acid^21^. They possess unique properties that make them attractive solvents for ODS. These properties include a low melting point, high thermal stability, non-flammability, and negligible vapor pressure^22–25^. DES also has the advantage of being customizable, low cost, and simple preparation, as their physical and chemical properties can be tailored by selecting specific components^26–28^. The use of DES in ODS offers several benefits^29–32^. They provide a suitable medium for the reaction, enhancing the solubility and availability of sulfur compounds^33–36^. DES can selectively extract sulfur compounds from the fuel matrix, facilitating their subsequent oxidation^37^. Furthermore, DES exhibit good stability and can be easily recycled, making them environmentally friendly and economically viable for practical applications^38–40^. Researchers continue to explore and optimize the use of DES in ODS processes, seeking to improve desulfurization efficiency, reduce costs, and address any challenges that may arise^41–44^. The development of DES-based systems for ODS holds promise for the production of cleaner fuels with significantly reduced sulfur content, contributing to environmental sustainability and compliance with stringent regulations.

In our research, our main focus was to develop a facile and straightforward deep eutectic solvent (DES) that could act as a catalyst and reaction medium in organic transformations^45–47^. Herein, we reported on the synthesis of a Fenton-like DES based on *N*-formyl morpholine and iron salts as a green solvent system that could serve as both a catalyst and a reaction medium for oxidative desulfurization while maintaining environmentally friendly conditions.

## Experimental

### General

The chemical compounds and solvents used in our research, including N-formyl morpholine (NFM), DBT, BT, MPS,FeCl_3_⋅6H_2_O, HCl 37%, commercial salts were purchased from supplier. To characterize the DES catalyst, various methods were analytical instrument were employed. FT-IR spectra were acquired using a Bruker Vector-22 infrared spectrometer with KBr disks, enabling the identification and characterization of chemical bonds and functional groups present in the samples. For elemental analysis, EDX (Energy-dispersive X-ray spectroscopy) studies were conducted using a scanning electron microscope (VEGA3 TESCAN) operating at 20 kV, providing quantitative information about the elemental composition of the samples. To determine the melting point of the compounds, a Büchi 535 melting point apparatus was utilized. This allowed for the measurement of the temperature at which the substances transitioned from solid to liquid phase, providing valuable information about their thermal properties. Thermal gravimetric analysis (TGA) was performed using a Netzsch-TGA 209 F1 instrument under a nitrogen atmosphere. The TGA data was obtained by subjecting the samples to a heating rate of 25 °C/min, ranging from room temperature to 850 °C. This technique enabled the study of the thermal stability and decomposition behavior of sample as a function of temperature. In addition, the sulfur content was determined by gas chromatography (Agilent 7890A) coupled with a flame ionization detector (GC-FID). Separation was achieved using an HP-5 capillary column with dimensions of 30m × 0.32mm inner diameter × 0.25 μm film thickness.

### Preparation of DES

The preparation of the DES involved two steps, as outlined below.

**Figure 2:**
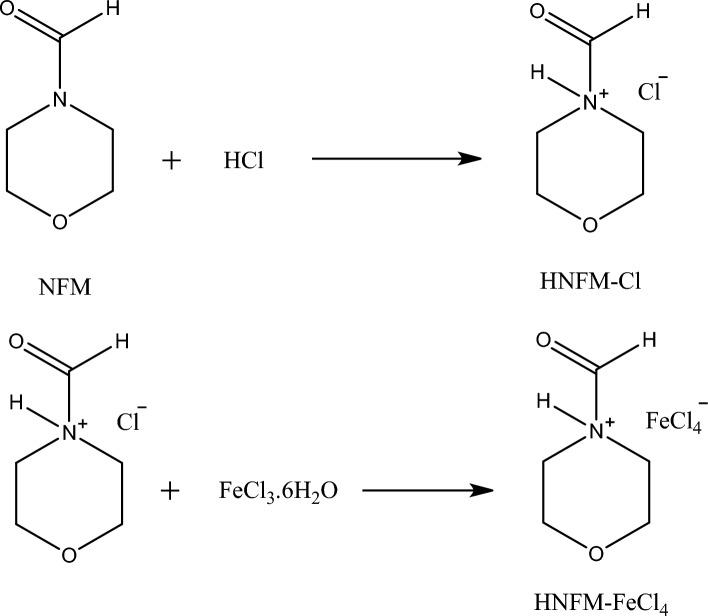
Preparation of novel DES.

In a round bottom flask, 100 mmol of *N*-formylmorpholine (NFM) and 100 mmol of HCl 37% were mixed and was heated under stirring at 50 °C for 24 h. The resulting mixture was then subjected to evaporation using a rotary evaporator until a white solid, HNFM-Cl, was formed. In the following steps, to the HNFM-Cl obtained from Step 1, 100 mmol of commercial salts (Table 1) for example FeCl_3_ was added. The mixture was further heated at 60°C and allowed to stir for 24 h and after that a homogeneous suspension was formed. Excess water was evaporated, yielding a desired DES (Fig. 2).

**Table 1:** Thermal properties data of DES in N_2_ atmosphere.

Sample	T_5_ (°C)	T_10_ (°C)	T_max1_ (°C)	T_max2_ (°C)	T_max3_ (°C)	T_max4_ (°C)	Char yield (%) (800 °C)
DES	223	243	302	332	415	616	7.5

### Extractive oxidative desulfurization procedure

The extractive oxidative desulfurization procedure was conducted using the following steps: first, the model oil containing DBT with a sulfur content of 250 ppm was prepared. For this propose 1.46 g of DBT dissolved in 1 litter of n-heptane and the DBT and n-heptane thoroughly were mixed until the DBT is completely dissolved. This model oil was used in subsequent extractive oxidative desulfurization processes. In a 50 mL two-necked round bottom flask containing a magnetic stirrer, 200 mg of the prepared DES (HNFM-FeCl_4_), 5 mL of the modal oil containing DBT and 30 wt% H_2_O_2_ were added to the flask at room temperature, respectively. This mixture was subsequently employed in extractive oxidative desulfurization experiments, serving as a representative system for evaluating the optimization of desulfurization processes. After the completion of a reaction, was subjected to analysis using gas chromatography (GC) and gas chromatography-mass spectrometry (GC–MS) spectroscopy.

## Result and discussion

**Figure 3:**
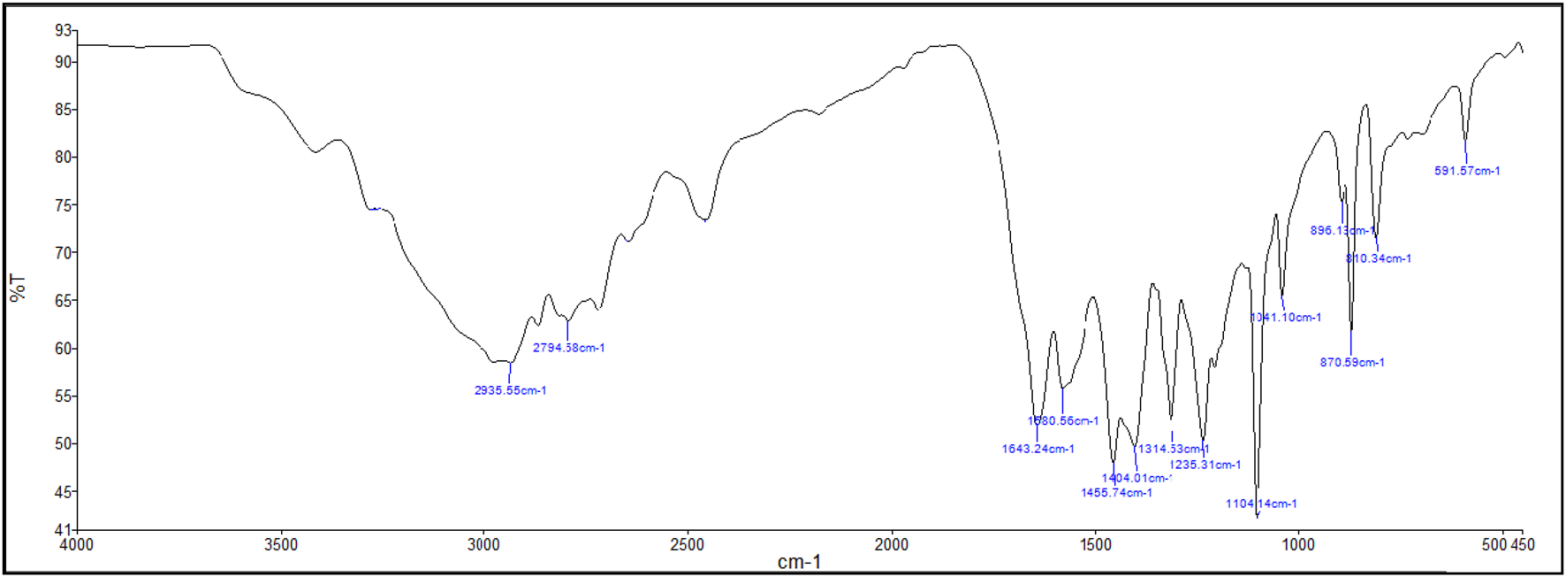
FTIR spectra of DES.

After synthesizing the desired DES using a straightforward method, it is crucial to characterize it using various spectroscopy and thermal analysis techniques. In the first, the DES was analysed using FTIR spectroscopy (Fig. 3), which revealed several significant absorption bands. One notable absorption occurs at approximately above 2935 cm^−1^, indicating the presence of a tertiary ammonium salt within the DES. Another absorption peak around 2935 cm^−1^ corresponds to C–H aliphatic stretching vibrations. The presence of an amidic carbonyl group is evident from a strong and sharp absorption band at approximately 1643 cm^−1^, which can be attributed to C=O stretching. The absorption band at around 1455 cm^−1^ is associated with methylene C–H bending vibrations. Additionally, a pronounced absorption band at 1104 cm^−1^ is observed, which can be attributed to C–O stretching.

**Figure 4:**
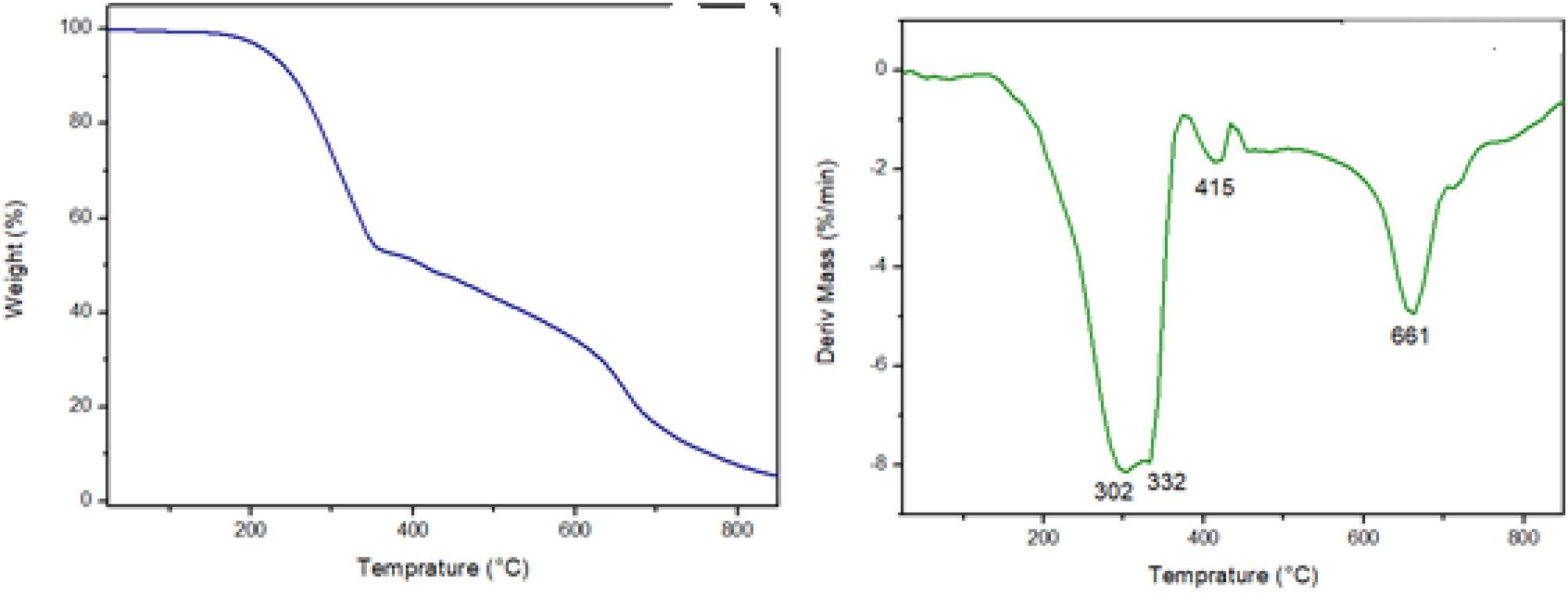
TGA (right) & and DTG (left) thermographs of DES.

The thermal properties of the deep eutectic solvent (DES) were investigated using the thermogravimetric analysis (TGA). The TGA results revealed three major mass losses, along with some minor losses, which confirmed the structural composition of the DES (as shown in Table 1 and Fig. 4). The minor mass loss observed at around 100 ºC can be attributed to the evaporation of water and other solvent components present in the DES. The first major mass loss occurs at 302 °C, indicating the N–C cleavage within the DES structure. This suggests the decomposition of the organic segment of the DES. The second major mass loss takes place at 332 °C, signifying the further decomposition and degradation of the organic constituents in the DES. The third and fourth mass losses occur at 415 °C and 661 °C, respectively. These mass losses are associated with the transformation of the FeCl_4_ anion to FeCl_2_ at 415 °C, and the subsequent formation of FeCl_2_ and Fe_3_C at 661 °C according to previous studies^21^. The residue char remaining at 800 °C was found to be 7.5%, confirming the presence of an organic–inorganic hybrid structure in the DES. Additionally, the stability of the DES (HNFM-FeCl4) was evaluated by subjecting it to increasing temperatures. The results, as shown in Table 1, indicated that only a 5% mass loss occurred until 223 °C, and a 10% mass loss was observed at 243 °C. After reaching temperatures higher than 243 °C, the DES (HNFM-FeCl4) started to decompose. These findings demonstrate the remarkable stability of the DES up to these temperatures, suggesting its suitability for applications that require thermal resilience.

**Figure 5:**
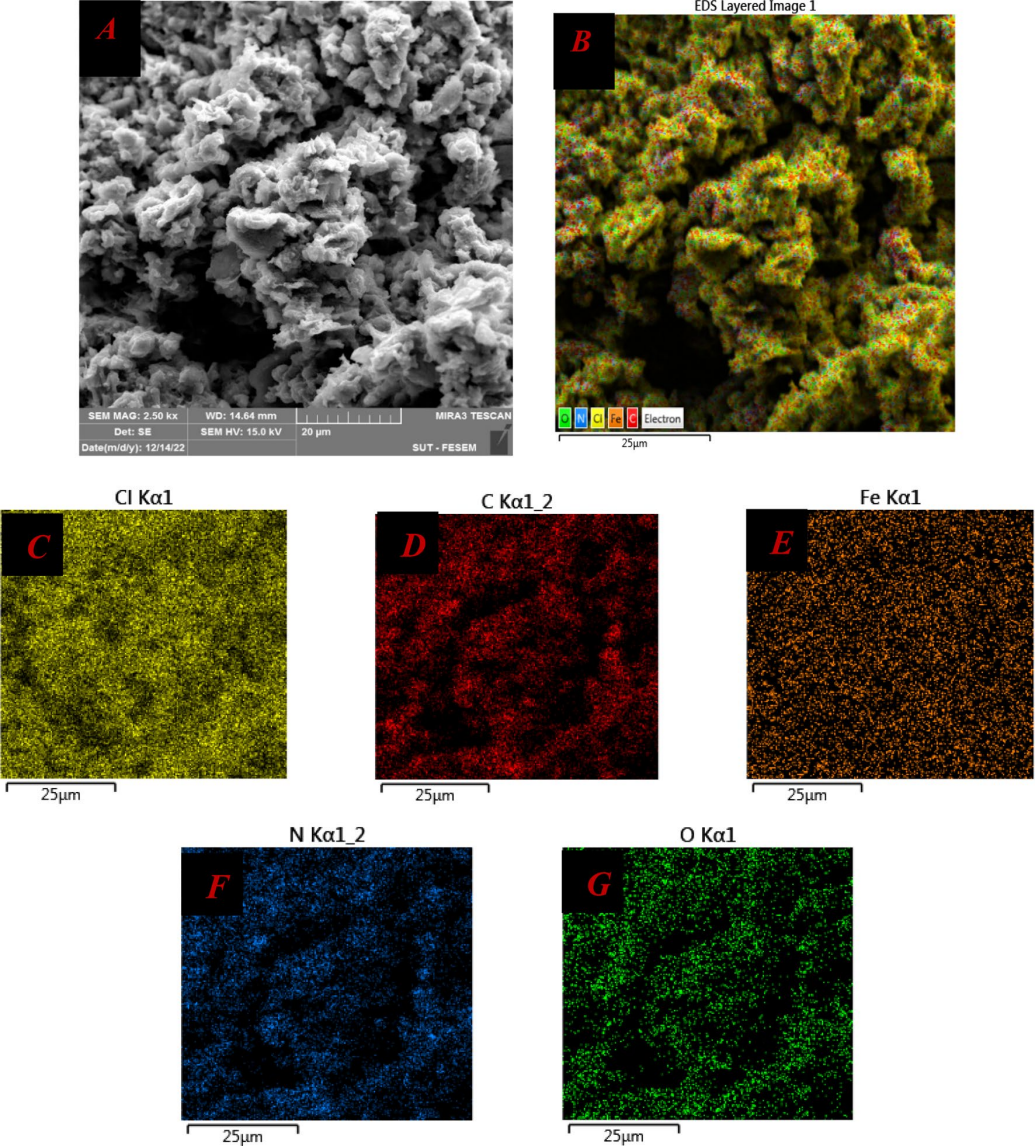
SEM image (**A**), element map accumulative (**B**), Cl map (**C**), C map (**D**), Fe map (**E**), N map (**F**), O map (**G**).

The surface morphology of the DES was investigated using SEM technique. The SEM analysis revealed a distinct and uniform surface vicissitude on the DES, indicating potential suitable sites for the progression of oxidation reactions. In addition to the SEM analysis, energy-dispersive X-ray spectroscopy (EDS) was performed to obtain elemental composition information (Table 2 and Fig. 5). The EDS image showed a homogeneous distribution of elements throughout the DES. The EDS statistical results confirmed that the weight percent of iron (Fe) in the DES matched the reported char yield and the expected mass present in the molecular formula. Furthermore, mapping images obtained from the EDS analysis demonstrated the homogeneity of the DES. These findings are presented in Table 2 and Fig. 5, providing visual evidence of the uniform distribution of elements within the DES. The combined SEM and EDS analysis contributes to a comprehensive understanding of the surface morphology, elemental composition, and homogeneity of the DES.

**Table 2:** EDS data of DES.

Element	Line type	Apparent concentration	k ratio	Wt%	Wt% sigma	Atomic %	Standard label
C	K series	1.96	0.01963	39.15	0.37	50.94	C Vit
N	K series	6.79	0.01209	30.26	0.41	33.76	BN
O	K series	0.80	0.00269	5.32	0.15	5.19	SiO2
Cl	K series	6.71	0.05868	18.82	0.16	8.30	NaCl
Fe	K series	1.92	0.01923	6.45	0.12	1.80	Fe
Total				100.00		100.00

Following the successful preparation and characterization of the DES, the optimization of the oxidation reaction was undertaken by exploring various reaction conditions. Several parameters were investigated to improve the efficiency of the process. These parameters included the selection of anion salt, temperature, catalyst quantity, oxidant type, oxidant-to-sulfur ratio, and reaction duration. By systematically studying these factors, the aim was to enhance the catalytic activity, selectivity, and overall performance of the process. In the initial step of optimization, various commercial salts, namely NiCl_2_, ZnCl_2_, SnCl_2_, CoCl_2_, CrCl_3_, SbCl_3_, MnCl_2_, FeCl_2_, FeCl_3_ and Fe(NO_3_)_3_ were used to prepare NMF based DES and results were shown in Table 3. Each DES was evaluated for its efficiency in oxidizing DBT in the organic substrate. The results obtained are summarized in Table 3, with the oxidation efficiencies of DBT being determined as follows: NiCl_2_ (56%), SnCl_2_ (43%), ZnCl_2_ (54%), CoCl_2_ (48%), CrCl_3_ (51%), SbCl_3_ (46%), MnCl_2_ (39%), Fe(NO_3_)_3_ (35%), FeCl_2_ (71%) and FeCl_3_ (85%). Based on these results, FeCl_3_ was identified as the most effective salt for oxidizing DBT, exhibiting a high oxidation efficiency of 90%. Therefore, FeCl_3_ was selected as the optimal salt for further experiments and process optimization.

**Table 3:** The effect of various metal salts based DES.


Entry	Salts	DES (salts: HNFM 1:1)	Sulfur removal %
1	NiCl_2_		56
2	SnCl_2_		43
3	ZnCl_2_		54
4	CoCl_2_		48
5	CrCl_3_		51
6	SbCl_3_		46
7	MnCl_2_		39
8	Fe(NO_3_)_3_		35
9	FeCl_2_		71
10	FeCl_3_		85

**Figure 6:**
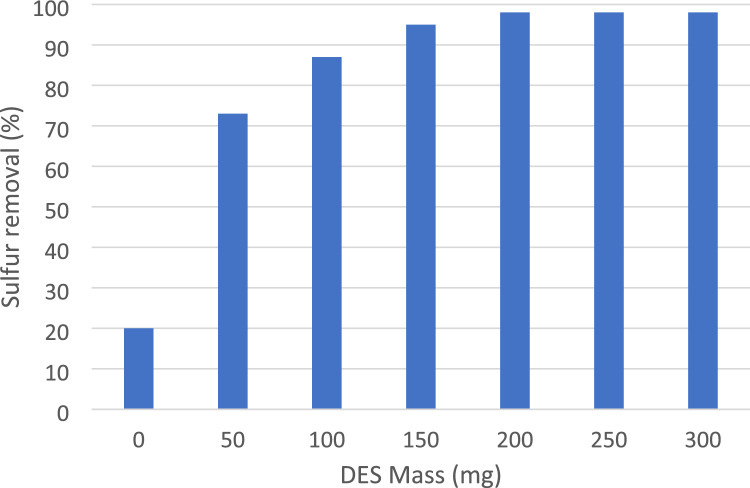
Optimization DES mass.

In the second step of optimization, the mass of the DES was investigated to determine its effect on the oxidation of DBT. Initially, without the DES, the yield of oxidation was only 7%, indicating the limited effectiveness of the reaction in the absence of the DES. To optimize the DES mass, different amounts of DES were loaded into the reaction system. Loading 50 mg of DES resulted in a significantly increased yield of oxidation (72%). Further increasing the DES mass to 100, 150, 200 250 and 300 mg led to yield of 85%, 95%, 98%, 98% and 98% respectively. However, upon further increasing the amount of DES, no significant improvement in the yield of oxidation was observed. These results, as summarized in Fig. 6, demonstrate the optimum DES mass was found to be 200 mg, which achieved a high yield of 98% oxidation. Beyond this point, increasing the DES mass did not provide any additional benefits in terms of improving the oxidation yield and times.

**Figure 7:**
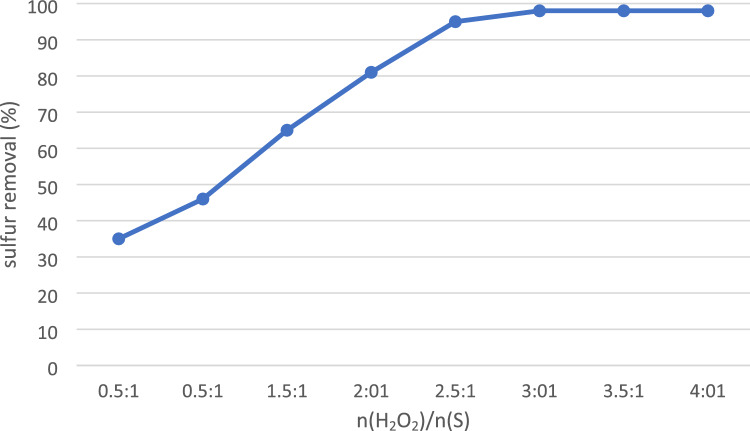
H_2_O_2_ optimization ratio to sulfur content.

In the optimization process, another important step involved optimizing the molar ratio of hydrogen peroxide (H_2_O_2_) to DBT and results were shown in Fig. 7. While stoichiometric ally, 2 mol of H_2_O_2_ are required to fully oxidize DBT to dibenzothiophene sulfone (DBTO_2_), it is important to consider that reactions may not proceed with 100% efficiency. Therefore, a higher molar ratio of reactants is often needed to ensure complete conversion. To determine the optimal H_2_O_2_ molar ratio to DBT, seven different ratios of n(H_2_O_2_)/n(S) including 0.5:1, 1:1, 1.5:1, 2:1, 2.5:1, 3:1, 3.5:1, and 4:1 were tested: The corresponding sulfur removal yields were found to be 35%, 42%, 65%, 81%, 95%, 98%, 98% and 98%, respectively. Interestingly, the results indicate a direct proportionality between the molar ratio of H_2_O_2_ to DBT (O/S ratio) and the sulfur removal yield. As the O/S ratio increased from 0.5:1 to 3:1, the sulfur removal yield steadily improved. However, beyond an O/S ratio of 3:1, there was no significant increase in the sulfur removal yield. These results suggest that an O/S ratio of around 3:1 is optimal for achieving high sulfur removal yields in the DBT oxidation reaction.

**Figure 8:**
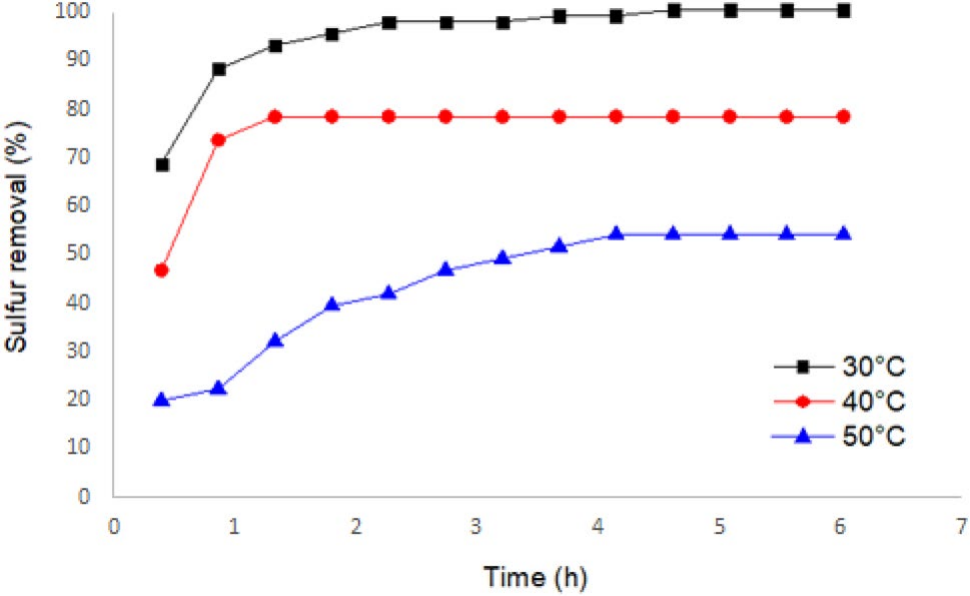
Temperature and Time optimization.

In the final step of optimization, the time and temperature conditions for the oxidation reaction were investigated (Fig. 8). Three different temperatures, 30 °C, 40 °C, and 50 °C, were tested to identify the most effective temperature for the reaction. The results indicated that the most effective temperature for developing the oxidation reaction was reported to be 30 °C, followed by 40 °C and 50 °C, respectively. It is important to note that at higher temperatures, there is a higher likelihood of hydrogen peroxide decomposition, which can contribute to a reduction in sulfur removal efficiency. In addition to temperature, the reaction time was also considered. It was observed that extending the reaction time beyond 2.5 h did not significantly increase the effectiveness of the oxidation process. Therefore, it can be inferred that the reaction reaches its maximum efficiency within this time frame. These findings suggest that the optimal temperature for the oxidation reaction is 30 °C, and it is recommended to keep the reaction time within a range of approximately 2.5 h.

**Figure 9:**
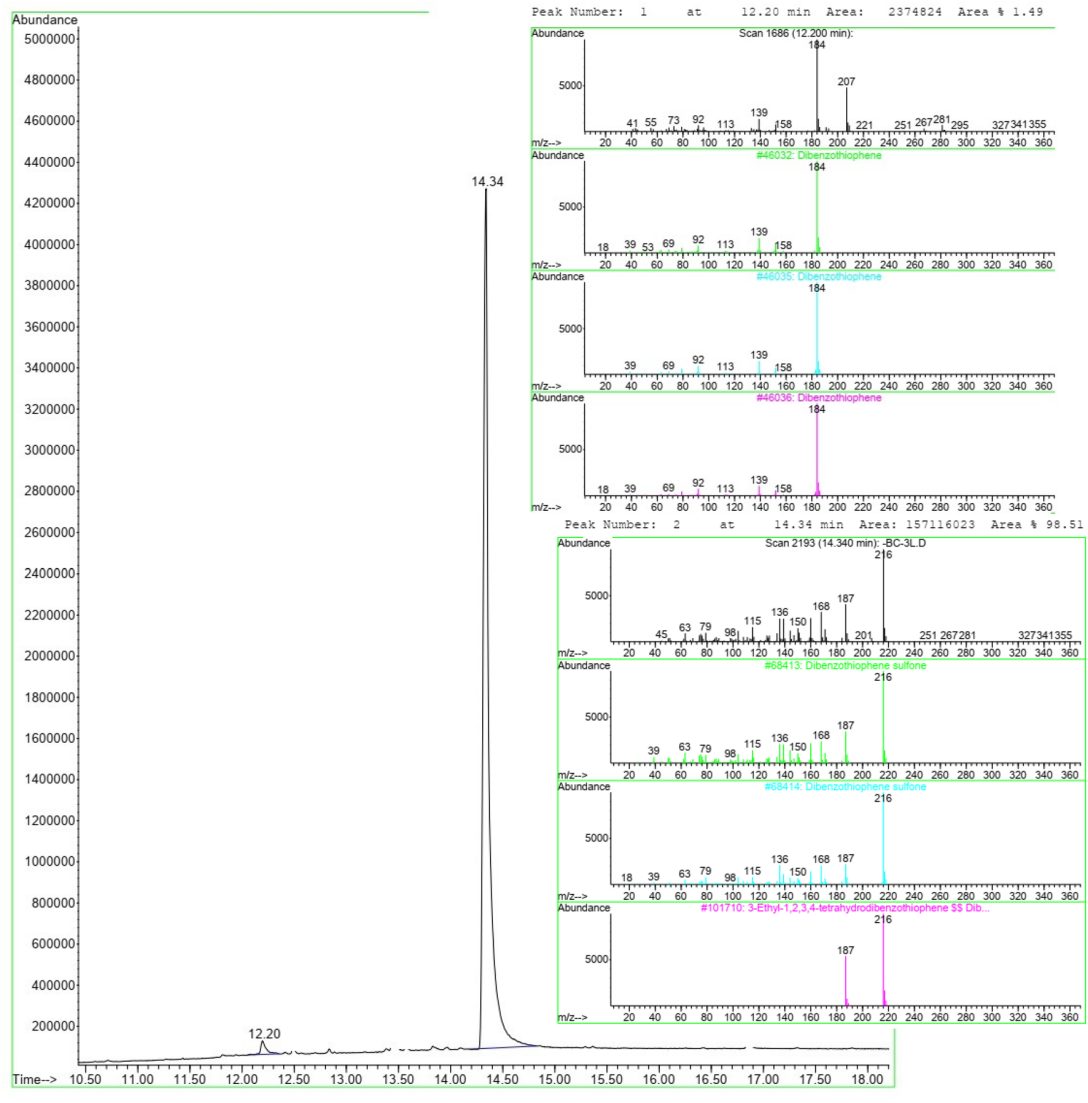
GC–MS analysis of reaction mixture under optimized conditions.

By subjecting the reaction mixture to GC–MS analysis, it is possible to assess its purity, detect any impurities or byproducts, and gain insights into its chemical composition. According to the GC–MS analysis conducted under optimized conditions, (Fig. 9) it was found that DBT was converted to dibenzothiophene sulfone with a yield of 98.51%.

Under optimized conditions, the reactivity of various sulfur-containing compounds, including benzothiophene (BT), 4,6-dimethyldibenzothiophene (4,6-DMDBT), and methyl phenyl sulfide (MPS), was examined in the oxidative desulfurization process. The results, as shown in Table 4, indicate that the order of sulfur removal was MPS, DBT, BT and 4,6-DMDBT.

**Table 4:** The sulfur removal for different sulfur compounds.

Entry	Name	structure	Time	Sulfur removal (%)
**a**	MPS		2.5	99
**b**	DBT		2.5	98
**c**	BT		2.5	94
**d**	4,6-DMDBT		5	49

**Figure 10:**
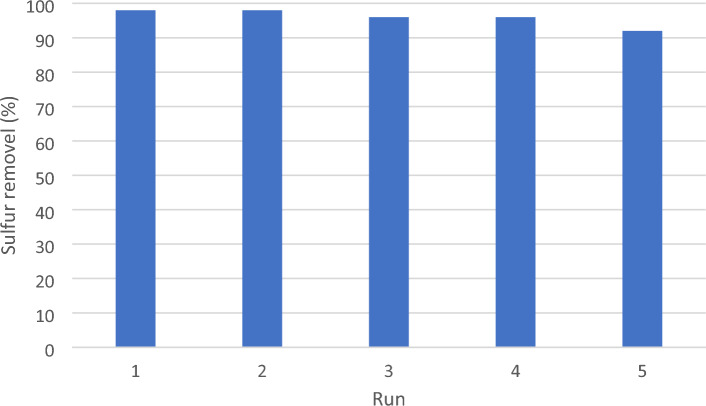
Reusability of DES.

In order to assess the reusability of the DBT extraction solvent and catalyst (DES) under optimized conditions, a scaled-up reaction was performed using 200 mg of DES. Once the reaction was completed, to separate the DES, ethyl acetate (10 mL) was added. The mixture was then subjected to centrifugation, allowing for the separation of the DES, which was subsequently washed with ethyl acetate (5 mL). After the washing step, the DES was dried. This process was repeated for four consecutive runs, and the results are presented in Fig. 10. The data obtained from the experiments demonstrated that the DES could be recycled and reused for at least five cycles without experiencing a notable decrease in yield, as depicted in Fig. 10.

**Figure 11:**
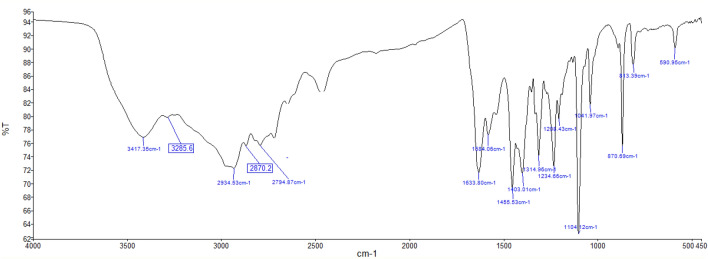
FTIR spectra of reused DES.

The fact that the reused DES did not showed any discernible differences compared to the fresh DES in the FTIR analysis, as depicted in Fig. 11, suggests that the DES remains stable in the desulfurization system. This stability is an encouraging finding as it indicates that the DES can be reused without significant degradation or alteration in its chemical composition and properties.

**Figure 12:**
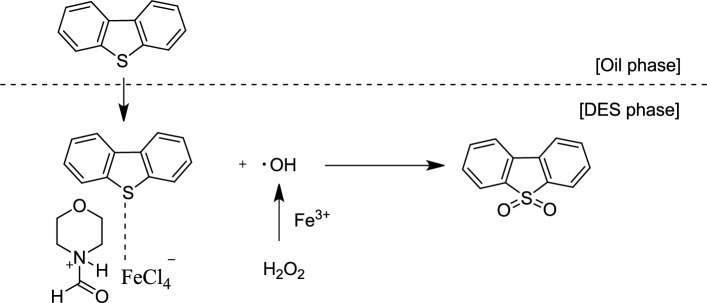
The proposed reaction mechanism.

Based on the literature^48–50^ and information in this article, a plausible mechanism has been proposed for this system (Fig. 12). Although the Fenton-like reagent (Fe^3+^/H_2_O_2_) has been widely used in organic oxidations, the exact mechanism of its action is still not fully understood. Considering that the DES solvent is immiscible in the model oil, the oxidation process is therefore a two-step process. In the first step, DBT is extracted in the DES phase, and in the next step, sulfur is oxidized in the sulfone. The DES acts similarly to the Fenton-like reagent and produces hydroxide radicals, which in the next step, cause the oxidation of sulfur to sulfoxide.

## Conclusion

In summary, this study investigated the application of Fenton-like DES as catalysts and reaction media for the oxidative desulfurization of organosulfur compounds contained in fuels at ambient temperature and atmospheric pressure. The various low cost metal salts based DES were designed and used as both catalyst and extractant for ODS process. Among the DESs studied, the iron-based DES, in combination with hydrogen peroxide as the oxidant, exhibited the highest catalytic activity for the oxidation of sulfur-containing compounds to sulfones. The DES system demonstrated stability and recyclability, rendering it suitable for practical implementation in fuel desulfurization processes. The ability to recover and reuse the DES enhances its economic viability and sustainability.

## Data Availability

All data generated during this study are included in this published article.
